# Theoretical justifications for using word-based stimuli in the implicit association test for physical activity

**DOI:** 10.3389/fpsyg.2026.1847599

**Published:** 2026-07-02

**Authors:** Yoongu Lee

**Affiliations:** Department of Physical Education, Dong-A University, Busan, Republic of Korea

**Keywords:** dual-process theory, implicit association test, implicit attitudes, physical activity, picture-based stimuli, word-based stimuli

## Abstract

Over the past decade, researchers have increasingly drawn on dual-process models to explain why individuals fail to translate physical activity intentions into sustained behavior. According to these models, a person’s behavior can be influenced by two different types of process; first, reflective processes (e.g., intention) and, second, automatic processes (e.g., habit). The IAT offers researchers a means of capturing automatic evaluative associations that self-report measures cannot access. However, the choice of either word-based or picture-based stimuli in physical activity IAT can meaningfully affect the sensitivity, reliability, and cross-cultural applicability of measurements. This article presents my perspective on the theoretical and methodological justification for recommending word-based stimuli as a reasonable default for assessing generalized implicit attitudes in physical activity IAT, drawing on empirical work in cognitive psychology, health behavior research, and exercise psychology. I argue that word-based stimuli may tend to produce more consistent response times, somewhat higher internal reliability, and lower interpretive variability than picture-based stimuli, while emphasizing that this evidence is largely indirect, deriving from studies that did not directly compare the two modalities within the same design. I then outline future applications of word-based IAT for advancing both understanding and intervention design in physical activity.

## 1 Introduction

The literature suggests that physical inactivity is among the leading preventable causes of morbidity and mortality worldwide (Guthold et al., 2018). The field of physical activity has been attempting for decades to provide interventions utilizing social cognitive theory-based programs to address the long-standing problem of low participation rates in regular physical activity. However, despite the numerous attempts by researchers and practitioners working within this area, it is clear that there are significant gaps between people’s intentions to be physically active and their actual behavior (Rhodes and de Bruijn, 2013). Additionally, meta-analysis has shown that the percentage of variance explained in physical activity behavior based on intention is small (Rhodes and de Bruijn, 2013); thus, there appears to be considerable amounts of variance in physical activity behavior that needs to be addressed.

To address this issue, researchers have begun looking beyond traditional reflective explanations toward the automatic determinants of physical activity. Dual process theories offer a way of conceptualizing this broader view. They posit two systems that jointly govern behavior: a reflective system, which supports purposeful thinking, goal setting, and planning, and an automatic system, which comprises learned associations, affective responses to stimuli, and cue-driven habits (Strack and Deutsch, 2004; Evans and Stanovich, 2013). On this account, even when individuals deliberately decide to be physically active, automatic evaluative processes are activated in parallel and can be triggered by environmental cues that override deliberate reasoning (Brand and Ekkekakis, 2018; Strobach et al., 2020).

The Physical Activity Adoption and Maintenance Model (Strobach et al., 2020; Jekauc et al., 2024) formalizes dual-process theory for physical activity, holding that explicit factors (intention, self-regulation, executive function) and implicit factors (affective evaluation, habit) contribute to maintenance through direct and indirect paths. During initial adoption, behavior is driven primarily by explicit factors; with continued engagement, however, it comes to depend increasingly on the degree to which the behavior has become habitual or has acquired positive valence at an automatic level. Long-term participation is therefore shaped less by what individuals intend or self-regulate than by how positively they evaluate physical activity at an implicit level (Rebar et al., 2016; Phipps et al., 2022).

The Implicit Association Test (Greenwald et al., 1998) has been the most commonly employed tool for assessing automatic evaluation associations in individuals. By employing reaction time data as an indicator of associative strength among abstract concepts and attribute categories, researchers can assess the degree to which individuals possess implicit evaluations of various attributes. An analysis of 26 studies consisting of 55 effect sizes found that there was a small statistically significant relationship between the implicit attitude toward physical activity and self-reported participation in physical activity (Chevance et al., 2019). Importantly, this relationship was independent of study design or sample characteristics, indicating that implicit attitudes represent a reliable, albeit moderate, predictor of physical activity behavior. Although there is now an expanding literature demonstrating the predictive validity of the physical activity IAT, relatively little attention has been directed toward one fundamental aspect of its design—namely, whether to use word-based or picture-based stimuli. In my view, this choice has important implications for measurement quality. The sections that follow examine how stimulus modality shapes reliability and generalizability in physical activity IAT research, and why this has been underappreciated in the existing literature.

## 2 Reflective and automatic processes in physical activity

Reflective processes refer to the cognitive structures as defined by social cognition theory which are used to determine why people may be willing to take on a specific action or behavior (Ajzen, 1991). Examples include attitude, subjective norms, perceived behavioral control, self-efficacy, and intention. These variables account for a large percentage of the variability in the expressed intentions of people to participate in physical activity; however, they do not ensure that individuals will act on those intentions (Rhodes and de Bruijn, 2013).

By contrast, the automatic process involves psychological mechanisms that operate quickly, effortlessly, and largely outside conscious awareness. Implicit attitudes refer to learned associations between concepts and affective valences (Greenwald and Banaji, 1995). Individuals who hold positive implicit attitudes toward physical activity tend to report higher affective responses during exercise and engage in more moderate-to-vigorous physical activity (de Oliveira Calado et al., 2022). Habits comprise cue-responses formed by repetition in stable environments, allowing relatively little cognitive involvement before initiating actions (Gardner, 2015; Wood and Rünger, 2016). When a habit is formed, the impact that an individual’s intention to perform the action has on whether they will act on their intention will decrease. In addition, as habits are formed, the environment becomes more in charge of initiating actions due to environmental cues (Rebar et al., 2016; Phipps et al., 2022). Phipps et al. (2023) reported reciprocal relationships between individuals’ habits and implicit attitudes toward the physical activity being performed. This relationship was shown to have been influenced by contextual disruptions caused by COVID-19 which further reduced the effect of explicit motivational intentions.

### 2.1 The case for measuring implicit processes

Self-report methods remain central to evaluating reflective constructs; however, they are ill-suited for measuring automatic associations. Individuals may exaggerate their exercise frequencies due to social desirability biases (Adams et al., 2005), and may not possess conscious access to the evaluative connections driving their spontaneous behavioral dispositions (Rebar et al., 2016). The IAT addresses these issues by using response latencies to measure how much more quickly people respond to stimuli associated with positive or negative emotions. Differences in response latencies when sorting stimuli paired with pleasant versus unpleasant attributes indicate the strength of an individual’s implicit associations (Greenwald et al., 2003). Studies have shown that both implicit attitudes toward physical activity measured through IAT are predictors of physical activity behavior in prospective studies where participants’ physical activity was assessed using devices (Conroy et al., 2010; Chevance et al., 2019; de Oliveira Calado et al., 2022). Single category versions of the IAT provide the researcher the ability to measure individuals’ absolute implicit evaluation of physical activities rather than require them to compare physical activities to one another. This provides the researcher greater flexibility in their use of the IAT (Karpinski and Steinman, 2006; Phipps et al., 2022). Although there have been some advances regarding the use of the IAT in assessing physical activity attitudes, there continue to be concerns regarding the psychometric properties of physical activity IATs, particularly since modality of stimulus contributes significantly to measurement quality for both reliability and cross-cultural generalizability.

## 3 Stimulus modality in the IAT: word-based versus picture-based differences in cognitive processing

### 3.1 Differences in cognitive processing

There are many differences in both form and in the cognitive demands for word-based versus picture-based IATs. Word-based IATs require much less mental processing than do picture-based IATs. For example, when a word such as “exercise,” “health,” or “fatigue” is presented, lexical access quickly retrieves a small group of related words (Collins and Loftus, 1975) which allows an individual to make a quick decision as to whether it fits into one of their predetermined categories. This retrieval process occurs directly, and most closely approximates the conceptual categories that are being evaluated using this IAT type. On the other hand, processing visual stimuli involves significantly longer chains of processing. In order to reach the point at which the observer can determine what category the item belongs to, they first need to determine what object has been visually represented. Once identified, they must also interpret all of the context surrounding the representation (including background environment, people or objects being depicted, and actions taking place), before resolving any ambiguities that exist within the depiction (Foroni and Bel-Bahar, 2010). This multi-step processing adds unwanted variance in response latencies and increases the likelihood of noise entering into what is otherwise a measure of automatic evaluation (Meissner and Rothermund, 2015).

### 3.2 Evidence supporting word-based stimuli

Several lines of evidence suggest that word-based IAT stimuli offer psychometric advantages over picture-based stimuli for assessing generalized implicit attitudes. Foroni and Bel-Bahar (2010) showed that word-based IATs produced significantly larger effect sizes than did picture-based IATs. They attributed the disparity primarily to differences in the level of representation at which stimuli are mapped to their respective categories. In situations where the representational fit between the stimulus and the category label is high—as is usually the case with words that directly name the target concept—the effects produced in an IAT are more robust. Meissner and Rothermund (2015) expanded upon this by demonstrating that the word-visual difference was due largely to modality-matching: when both target and attribute stimuli were of the same modality (e.g., all-verbal IAT), recoding processes that can add or subtract variance in IAT scores were minimized.

Within the broader IAT literature, Axt et al. (2021) analyzed 64 evaluatively loaded attribute words across 13 IATs administered to over 60,000 participants and determined that nearly all positively and negatively valenced words provided acceptable-quality measurement. This finding suggests that selecting clearly evaluatively loaded words as attribute categories for IATs is sufficient to obtain robust measurement. This stands in contrast to the varying degrees of interpretation required to respond to exercise-related picture-based stimuli, which may include representations of specific activities, environments, or body types that carry idiosyncratically positive or negative evaluative meanings depending on who views them.

I contend that Construal Level Theory provides additional theoretical justification for distinguishing between word-based and picture-based IAT stimuli. Carnevale et al. (2015) demonstrated that words elicit high-level construal processing that identifies central, goal-relevant characteristics, while visual stimuli elicit low-level construal processing that focuses on peripheral, idiosyncratic aspects. Within a physical activity IAT paradigm, this means that a word stimulus (e.g., “exercise”) directs attention to an abstract conception of the concept, and not to peripheral perceptual attributes of the stimulus. By contrast, a visual stimulus depicting a person jogging could convey information regarding weather, terrain, level of physical exertion, and social context—each of which may be valued differently by different individuals. In my assessment, word-based IATs therefore tend to evaluate participants’ attitudes toward the concept of exercise rather than reactions to peripheral characteristics of the visual stimulus.

### 3.3 Cross-cultural and cross-contextual considerations

It is also important to consider that word-based stimuli are not culturally neutral by default. As indicated previously, the semantic range, affective value, and arousal implication of words vary significantly from culture to culture and from language to language. These cross-linguistic differences have direct implications for the validity of physical activity IATs in cross-cultural research. Core terms such as “exercise,” “sports,” “physical activity,” and “workouts” have no clean one-to-one translations across languages, and meaningful variability can arise even within a single language. The Korean terms 운동 (undong), 체육 (cheyuk), 스포츠 (seupocheu), and 헬스 (helseu), for instance, are all plausible renderings of “physical activity” or “exercise,” yet each evokes a distinct semantic field; selecting 운동 rather than 헬스 as a target stimulus would therefore sample a different construct. Such differences are easily missed by back-translation, which establishes surface equivalence but not the conceptual equivalence that valid stimulus selection requires (Brislin, 1970; Van de Vijver and Tanzer, 2004).

Word-norm research concerning affective words further supports these concerns. Affective norms for English words (ANEW; Bradley and Lang, 1999) was adapted across Portuguese, Italian, German, and Spanish samples. Although ANEW showed reasonable consistency in valence ratings across languages, consistent large differences were shown in rating arousal and dominance ratings (Soares et al., 2012; Montefinese et al., 2014; Schmidtke et al., 2014; Sarli and Justel, 2021). Further support comes from direct bilingual IAT studies. Studies comparing mean implicit associations across languages for the same bilingual participants showed that mean implicit association differences exceeded d > 0.70 (Ogunnaike et al., 2010).

**Figure 1:**
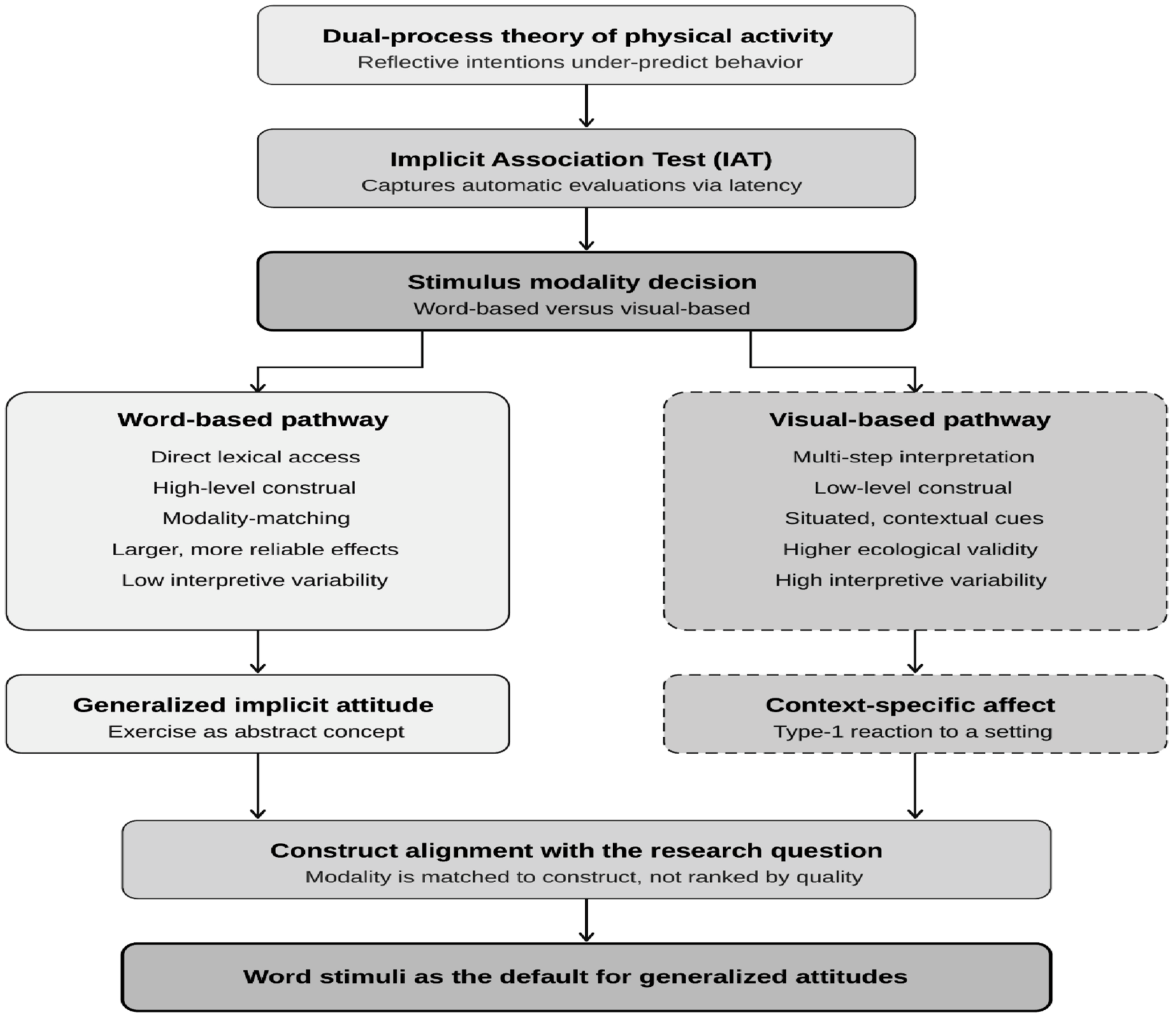
Conceptual framework for stimulus modality selection in the physical activity IAT.

Therefore, cross-cultural use of word-based PA-IAT stimuli should be accompanied by a multi-step validation process. Minimally, this should include free recall norming (Rebar et al., 2016), local valence ratings and arousal ratings, cognitive interviews to reveal unintentional connotations associated with each item, and pilot testing with reaction-time-based screening to remove poorly performing examples (Greenwald et al., 2022) (see Figure 1; Table 1).

**Table 1:** Conceptual comparison of word-based and picture-based stimuli for physical activity IATs with supporting evidence.

Dimension	Word-based stimuli	Picture-based stimuli	Key supporting references
Cognitive processing	Direct lexical access; constrained semantic activation	Multi-step: object identification → context interpretation → ambiguity resolution	Collins and Loftus (1975), Foroni and Bel-Bahar (2010)
Effect sizes	Evidence suggests larger, more robust effects	Evidence suggests smaller effects; susceptible to representational mismatch	Foroni and Bel-Bahar (2010), Meissner and Rothermund (2015)
Internal reliability	Potentially higher; recoding variance minimized when modality-matched	Potentially lower; modality mismatch may introduce additional variance	Bluemke and Friese (2008), Meissner and Rothermund (2015), Chevance et al. (2017)
Construal level	High-level: abstract, goal-relevant features	Low-level: peripheral, idiosyncratic features	Carnevale et al. (2015)
Cross-cultural applicability	Translatable via back-translation and semantic equivalence testing	Culture-specific visual associations; difficult to standardize	Ogunnaike et al. (2010), Rebar et al. (2016), Greenwald et al. (2022)
Interpretive variability	Likely lower; words directly name target concepts	Likely higher; picture-based stimuli carry contextual information (weather, terrain, body type, social context)	Foroni and Bel-Bahar (2010); Cope et al. (2018)
Ecological validity	Likely lower; abstracted from situated exercise cues encountered in daily life	Likely higher; replicates real-world environmental triggers of type-1 automatic responses	Antoniewicz and Brand (2014), Conroy and Berry (2017), Brand and Ekkekakis (2018)

A note on the scope and selection of evidence is warranted here. This article is intended as a Perspective rather than a systematic review, and the studies summarized in Table 2 were therefore selected to illustrate the theoretical arguments developed above rather than through an exhaustive or pre-registered search. I chose studies that are representative of each stimulus modality and of the predictive-validity literature on physical activity IATs, prioritizing work that reports the psychometric properties most relevant to my argument (internal consistency, test–retest reliability, effect size, and prospective prediction of behavior). Because this selection is illustrative rather than comprehensive, I do not attempt to estimate a pooled effect or to weight studies by quality, and I acknowledge that a different selection could place somewhat different emphasis on particular findings. What the assembled evidence does make visible, however, is a structural feature of the field that does not depend on which representative studies are chosen: word-based and picture-based IATs have rarely, if ever, been compared head-to-head within the same sample and design (Zenko and Ekkekakis, 2019). It is against this backdrop of indirect, cross-study evidence that the present recommendations should be read.

**Table 2:** Structured summary of representative physical activity IAT studies by stimulus modality.

Study	Modality	Format	Internal consistency	Test–retest reliability	Effect size	Predictive validity
Conroy et al. (2010)	Word	SC-IAT	—	—	*β* = 0.15	Yes (pedometer, 1 wk)
Hyde et al. (2012)	Word	SC-IAT	*α* = 0.63–0.73	*r* = 0.22 (1 wk)	*β* = −0.15 to −0.16	Yes (negative AE subgroup)
Chevance et al. (2017)	Word	IAT/SC-IAT	*α* = 0.64–0.97	ICC = 0.20–0.78	—	—
Rebar et al. (2016)	Word	SC-IAT	Split-half = 0.73–0.84	—	*β* = 0.24	Yes (accelerometer, 2 wk)
Phipps et al. (2023)	Word	ST-IAT	*α* = 0.63–0.71	*β* = 0.27 (stability)	*β* = 0.17	Yes (2 wk. follow-up)
Markland et al. (2015)	Picture	IAT	*ρ* = 0.91–0.93	—	*d* = 0.39	—
Antoniewicz and Brand (2016)	Picture	ST-IAT	—	—	η^2^part. = 0.11 (S1); η^2^part. = 0.29 (S2)	Yes (Study 2; ergometer)
Pfeffer and Strobach (2021)	Picture	ST-IAT	—	—	*β* = 0.16	Yes (IAT × Intention × Inhibition)

SC-IAT, Single-Category Implicit Association Test; ST-IAT, Single-Target Implicit Association Test; AE, Automatic Evaluation; S1/S2, Study 1/Study 2. Dash (—), not reported or not applicable.

### 3.4 Ecological validity and construct-level distinctions of picture-based stimuli

While word-based stimuli present numerous psychometric benefits, it is too early to describe picture-based stimuli as universally inferior. The Affective-Reflective Theory (ART; Brand and Ekkekakis, 2018) holds that the type-1 automatic affective reaction is triggered by perceptually specific, situated exercise cues that individuals encounter in daily life—precisely the kind of cues that picture-based IATs reinstate. On this account, picture-based stimuli hold a genuine theoretical advantage for indexing the type-1 process as ART defines it, because they reinstate the contextual cue that elicits the automatic affective response, whereas word-based stimuli necessarily abstract away from it. This lends picture-based IATs an ecological validity that abstract word-based stimuli cannot fully achieve (Conroy and Berry, 2017; Schinkoeth and Antoniewicz, 2017).

There is empirical support for this perspective. Antoniewicz and Brand (2014) demonstrated that automatic positive associations towards exercise settings were setting-specific. Non-fitness center exercising subjects failed to demonstrate a positive association with fitness center pictures - something that abstract word-based stimuli would not have masked. Cope et al. (2018) found that differences in image content resulted in significant variations in automatic exercise associations (*d* = 0.61 [outdoor > indoor]; *d* = 0.60 [sport > gym]) and Schinkoeth et al. (2019) found a relationship between picture-based assessments of exercise and heart rate variability, which provides physiologic evidence that visual stimuli elicit responses from somatic systems. For example, for individuals with low reading abilities such as young children, picture-based tasks are not only easier to complete but also more representative of their environment (Limmeroth and Raboldt, 2022). Taken together, these considerations suggest that the two modalities are best understood as indexing partially different constructs rather than as competing measures of the same construct. A word-based IAT primarily captures the automatic evaluation of physical activity as an abstract concept, whereas a picture-based IAT primarily captures the automatic affective reaction to a specific depicted context. Framed in this way, the choice of modality is not a question of measurement quality but of construct alignment with the research question. Accordingly, I recommend word-based stimuli as a reasonable default for assessing generalized implicit attitudes toward physical activity, while picture-based stimuli remain preferable when the research question concerns automatic evaluations of a specific setting, context, or moment—including studies that explicitly aim to operationalize ART’s type-1 process.

## 4 Discussion

In addition to the evidence discussed above, word-based stimuli can serve as a defensible default for Physical Activity IATs when the goal is to assess generalized implicit attitudes, a recommendation that has received little explicit attention in the literature to date. However, this does not constitute a “blanket” recommendation. It is recommended primarily for those conducting research into the assessment of general implicit attitudes towards physical activity as a whole, across diverse cultural or linguistic groups, where long-term study designs require high levels of test/re-test reliability, or in which participant exercise history varies significantly and therefore could lead to individual interpretation of image stimuli.

Conversely, there will be many research contexts in which picture-based stimuli will prove to be the more logical representation. Therefore, researchers should not feel constrained to use word-based representations unless they have sufficient reason to do so based upon their research questions. Research into automatic responses to particular exercise environments, or to particular aspects of exercise that relate to the body or sports related areas, are examples of such research. These would best be represented through picture-based stimuli, due to their ability to depict relevant information about these issues in greater detail than words alone. Similarly, research conducted with children or low literacy individuals may benefit from the use of picture-based stimuli as reading requirements may impact both latency and accuracy of response. Finally, interventions utilizing imagery/conditioning based paradigms may find theoretical coherency to utilize picture-based stimuli for both measurement and manipulation purposes (Antoniewicz and Brand, 2016; Markland et al., 2015). For assessing generalized implicit attitudes, word-based stimuli may offer greater conceptual clarity, more consistent reaction times, and less susceptibility to individual and cultural variability in interpretation; the available evidence, though largely indirect, also suggests they may afford higher internal reliability—properties that could contribute to more precise measurement. These properties are congruent with dual-process theories which demand clear differentiation between reflective and automatic contributions to behavior.

These recommendations carry important implications for designing interventions aimed at changing physical activity behavior. If word-based IATs can accurately assess changes in implicit attitudes over time, they provide valuable process measures for determining whether interventions targeting automatic processes change implicit attitudes before or alongside corresponding behavioral changes (Antoniewicz and Brand, 2016). Conditioning interventions that pair positive cues with physical activity cues should result in measurable shifts in IAT-assessed implicit attitudes. Similarly, habit-forming interventions that emphasize consistent pairing of behaviors with contexts could be assessed not only through behavior change but also through shifts in implicitly activated associations. The measurement stability of word-based designs over repeated administrations makes them particularly suited for longitudinal process evaluation.

I acknowledge that picture-based stimuli may retain value in certain research contexts. For example, examining how specific environmental features (design of park trails, facility aesthetic) are associated with positive or negative emotions related to exercise may require picture-based stimuli that cannot be captured by words alone. However, if picture-based stimuli are to be used as part of an IAT protocol, then it is necessary to utilize rigorous stimulus-validation procedures (including cross-cultural norming and minimal background conditions) so as to limit extraneous visual information (Foroni and Bel-Bahar, 2010). If the ultimate objective is accurate assessment of global implicit attitudes toward physical activity, well-developed word-based designs will generally be more appropriate.

Looking ahead, four directions merit particular attention. First, and most fundamentally, direct within-sample comparisons of word- and picture-based physical activity IATs are needed. Administering both modalities to the same participants under matched conditions—and relating each to prospective physical activity behavior—would make it possible to test, rather than infer, whether the two modalities capture partially distinct constructs, and would provide the head-to-head evidence that the present literature lacks. Second, developing universally standardized word-based stimulus sets for physical activity IATs would greatly aid in facilitating meaningful cross-study comparison and meta-analytic synthesis. Such sets would need to be tested for semantic equivalence in multiple languages and subjected to tests for evaluative stability across diverse populations. Axt et al. (2021) serves as a useful prototype for achieving this aim. Third, combining word-based IATs with ecological momentary assessment paradigms and wearable sensors would allow researchers to determine how implicit attitudes interact with moment-by-moment environmental and physiological contexts to influence decisions regarding physical activity engagement (Jekauc et al., 2024). Fourth, researchers utilizing interventions designed to modify automatic processes (evaluative conditioning, implementation intention interventions) should include IAT assessments as process measures to determine whether their programs induce changes in implicit attitudes concurrent with observed changes in behavior. These directions will be most productive if anchored in measurement tools with adequate reliability and in study designs that compare modalities directly rather than across separate samples.

## 5 Conclusion

The central argument of this Perspective is not that word-based stimuli are psychometrically superior to picture-based stimuli in any absolute sense, but that the two modalities appear to capture partially distinct constructs. A word-based IAT primarily indexes the automatic evaluation of physical activity as a generalized concept, whereas a picture-based IAT primarily indexes the automatic affective reaction to a specific, situated exercise context. Recognizing this distinction reframes the choice of stimulus modality as a question of alignment between the measure and the research question, rather than one of measurement quality. On this view, word-based stimuli are a reasonable default when the goal is to assess generalized implicit attitudes—particularly in cross-linguistic or longitudinal designs—while picture-based stimuli remain preferable when the research question concerns automatic responses to specific contexts, including those that operationalize the type-1 process of the Affective-Reflective Theory. Because the evidence currently available is largely indirect, this conceptual distinction should be regarded as a framework to be tested rather than an established conclusion. Direct, within-sample comparisons of word- and picture-based physical activity IATs—administering both modalities to the same participants under matched conditions—represent the most important next step, as they would allow the proposed construct-level distinction to be examined empirically rather than inferred across studies.

## Data Availability

The original contributions presented in the study are included in the article/supplementary material, further inquiries can be directed to the corresponding author.
